# A case report: pseudoxanthoma elasticum diagnosed based on ocular angioid streaks and the curative effect of Conbercept treatment

**DOI:** 10.1186/s12886-021-02069-0

**Published:** 2021-08-23

**Authors:** Chaoxiong Cui, Zhanyu Zhou, Yi Zhang, Ding Sun

**Affiliations:** 1 School of Clinical Medicine, Weifang Medical University, Weifang, Shandong China; 2 Department of Ophthalmology, Qingdao Municipal Hospital, Qingdao, Shandong China

**Keywords:** Pseudoxanthoma elasticum, Angioid streaks, Choroidal neovascularization, Bruch’s membrane, Conbercept

## Abstract

**Background:**

This article is a case report of pseudoxanthoma elasticum (PXE) which was diagnosed based on significant angioid streaks (AS) with choroidal neovascularization (CNV) and regain normal visual function by intravitreal injection with Conbercept.

**Case presentation:**

A 51-year-old woman was referred to the Ophthalmology Department of Qingdao Municipal Hospital (Qingdao, China) on September 14, 2020 for metamorphopsia and loss of vision in the left eye in the preceding three days. Past history: high myopia for more than 30 years, best corrected visual acuity (BCVA) of both eyes was 1.0 (5 m Standard Logarithm Visual Acuity chart in decimal notations), hypertension for six years, and cerebral infarction two years ago, no history of ocular trauma or surgeries or similar patients in family was documented. We used methods for observation, including fundus examination, optical coherence tomography (OCT), fluorescein angiography combined with indocyanine green angiography (FFA + ICGA). Due to her symptoms and manifestations, along with the appearance of her neck skin, which resembled ‘chicken skin’, we speculated that she should be further examined at the Department of Dermatology by tissue paraffin section and molecular pathology analyses, and the diagnosis of PXE was then confirmed. After intravitreal injection with Conbercept (10 mg/ml, 0.2 ml, Chengdu Kanghong Biotechnologies Co., Ltd.; Chengdu, Sichuan, China) she regained her BCVA.

**Conclusions:**

This patient regained her best corrected visual acuity through intravitreal injection with Conbercept. To the best of our knowledge, no publications are available on cases in which a vision loss and the normal visual function can be reverted by intravitreal injection with Conbercept. Although PXE is a disease with low incidence and thus no effective cure established, targeted symptomatic treatment can effectively retard the disease progression and improve visual function, such as intravitreal injection with Conbercept.

## Background

Pseudoxanthoma elasticum (PXE) is an autosomal recessive genetic disease that leads to progressive mineralization of the calcium compounds in the elastic fibers of the connective tissues, which results in adverse ocular changes and manifestations [1, 2]. The main characteristic of fundus imagery examination is angioid streaks (AS), which is the fracture of Bruch’s membrane (BM). In this case, we administered several intravitreal injections with Conbercept to revert the development of secondary choroidal neovascularization (CNV) of PXE and achieved outstanding results. Conbercept is a 143 kDa recombinant fusion protein which was composed of the second immunoglobulin (Ig) domain of vascular endothelial growth factor receptor-1 (VEGFR-1) and the third and fourth Ig domain of vascular endothelial growth factor receptor-2 (VEGFR-2) to the constant region of human IgG1, and it was designed as a receptor decoy with high affinity for all members in vascular endothelial growth factor receptor (VEGF) family, which includes VEGF-A, −B, −C, −D and placental growth factors (PlGF) [3–6]. Compared to Ranibizumab, the half-life of Conbercept is longer than Ranibizumab in the rabbit eyes although the intravitreous half-life of Conbercept in humans has not been reported yet [6]. In addition, Conbercept could induce a higher reduction of VEGF plasma levels and central retinal thickness while as safety and efficacy as Ranibizumab [6, 7]. The above-mentioned concludes that, Conbercept is a safe and effective anti-vascular endothelial growth factor (anti-VEGF) drug which could exert potent anti-angiogenic and anti-tumor effects due to the inhibitory effects to VEGF in vitro and vivo, and proved to be a promising option for the treatment of CNV [3, 7]. To the best of our knowledge, this article may be the first to provide evidence that CNV which is secondary to PXE could be reverted by an intravitreal injection with Conbercept [8–13]. In conclusion, typical ocular AS with skin manifestations were used in the primary diagnose of PXE and intravitreal injection with Conbercept may be an effective alternative treatment for the further therapy of secondary CNV caused by PXE.

## Case presentation

**Fig. 1:**
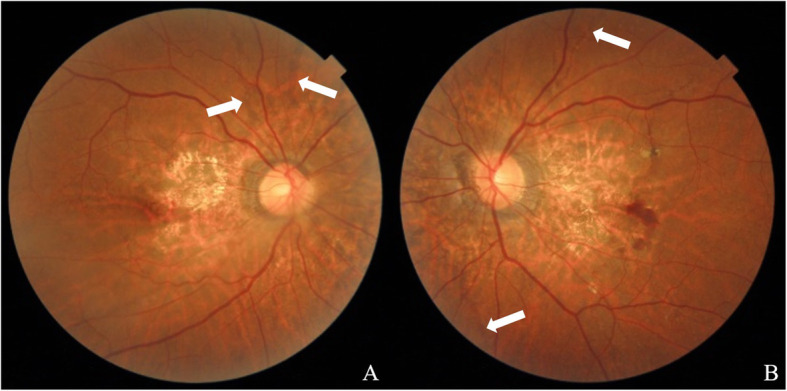
Fundus color photos taken on September 14, 2020. (**A**) Right eye; (**B**) Left eye. The optic discs of both eyes are clear with a light red color; C/D = 0.2, AS present radially around the optic discs of both eyes. The retina of the posterior pole area of the right eye is thinner than that of the other areas, and splinter hemorrhage is visible around the macular area. The retina of the posterior pole area of the left eye is thinning, and splinter hemorrhage can be found at lower areas of the macular area. The white arrows are pointing AS

**Fig. 2:**
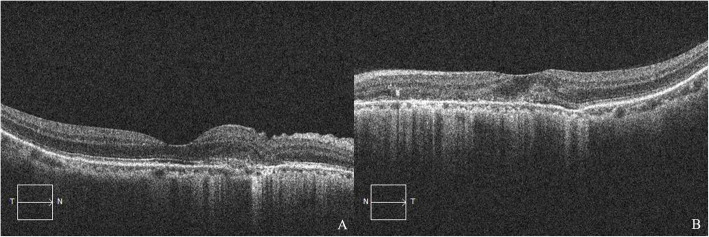
2020-9-14 OCT. (**A**) Right eye; (**B**) Left eye. (**A**): The spindle-like, hyperreflective lesion could be seen under the neuroepithelial layer of the nasal side of the fovea; (**B**): The normal structure of macular fovea had disappeared, and a spindle-like, hyperreflective lesion was observed

**Fig. 3:**
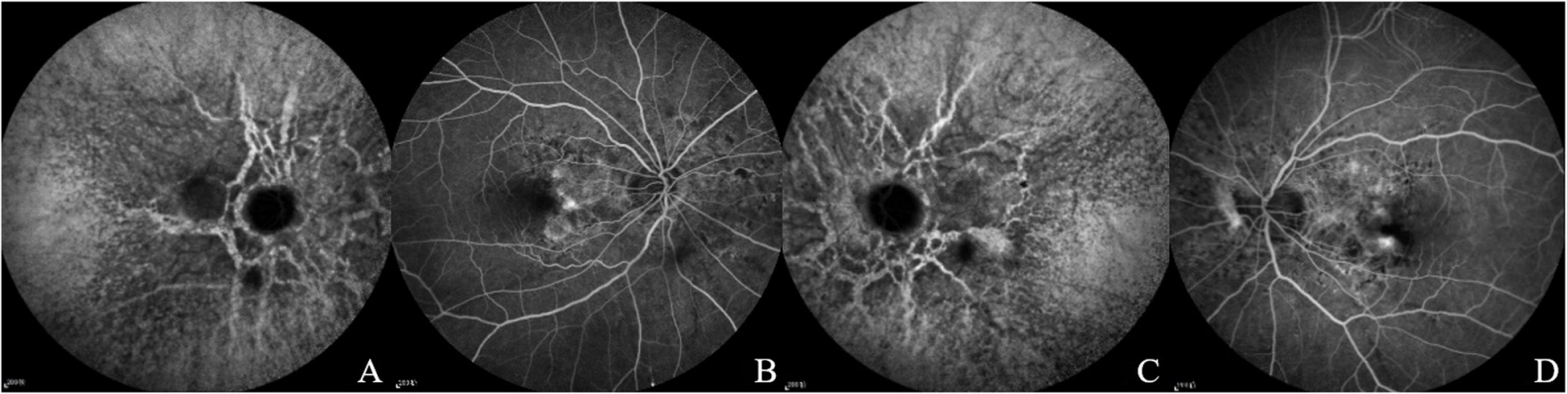
2020-9-14 FFA + ICGA. (**A**) and (**B**) show the right eye, while (**C**) and (**D**) show the left eye; (**A**) and (**C**): In the late stage of bilateral angiography, presented radial hyperfluorescence around the optic disc which represent AS; (**B**) and (**D**): The hyperfluorescence of the macular area is the leakage area of CNV

A 51-year-old woman was referred to the Ophthalmology Department of Qingdao Municipal Hospital (Qingdao, China) on September 14, 2020 for metamorphopsia and loss of vision in the left eye in the preceding three days. Past history: high myopia for more than 30 years, best corrected visual acuity (BCVA) of both eyes was 1.0 (5 m Standard Logarithm Visual Acuity chart in decimal notations), hypertension for six years, and cerebral infarction two years ago, no history of ocular trauma or surgeries or similar patients in family was documented. On ocular examination, the uncorrected distance visual acuity (UCDVA) of the right eye was 0.03 and BCVA was 0.8^+ 2^ (− 7.75DS/− 0.50 DC × 85°), counting fingers (CF)/20 cm in the left eye and BCVA was 0.4^+ 1^ (− 7.50DS); intraocular pressure (IOP) = 19 mmHg in both eyes (1 mmHg = 0.133 kPa), with no abnormalities in the anterior segments in both eyes. Fundus examination revealed radial angioid streaks (AS) around the optic discs of both eyes, subretinal hemorrhages were also visible below the macular area in her left eye (Fig. 1). Optical coherence tomography (OCT) showed choroidal neovascularization in both eyes (Fig. 2). Fluorescein angiography combined with indocyanine green angiography (FFA + ICGA) detected hyperfluorescence around the optic discs, and choroidal neovascularization leakage areas of both eyes were evident (Fig. 3). We supposed that these manifestations were consistent with the changes of the PXE fundus — AS.

**Fig. 4:**
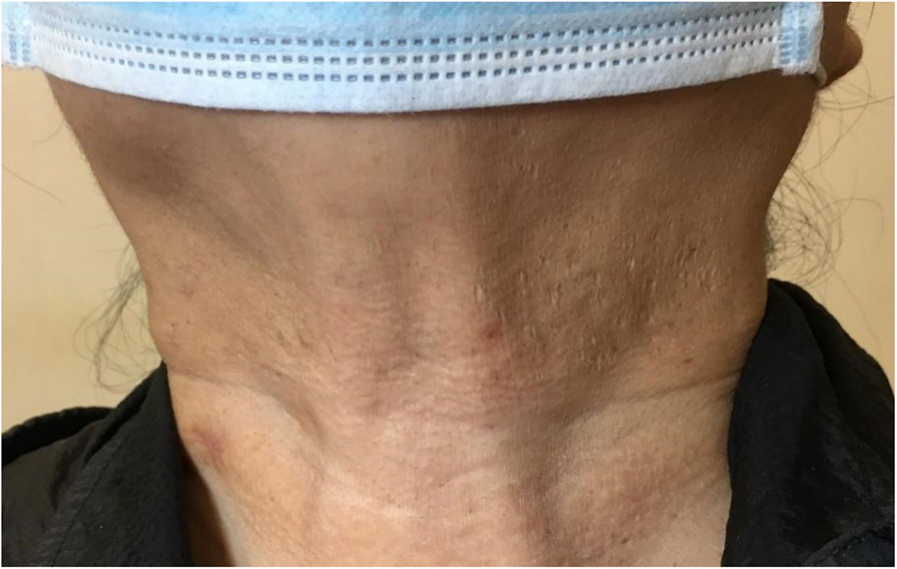
Photo of the neck skin. Yellowish papules are visible on the skin of the patient’s neck that resemble ‘chicken skin’

**Fig. 5:**
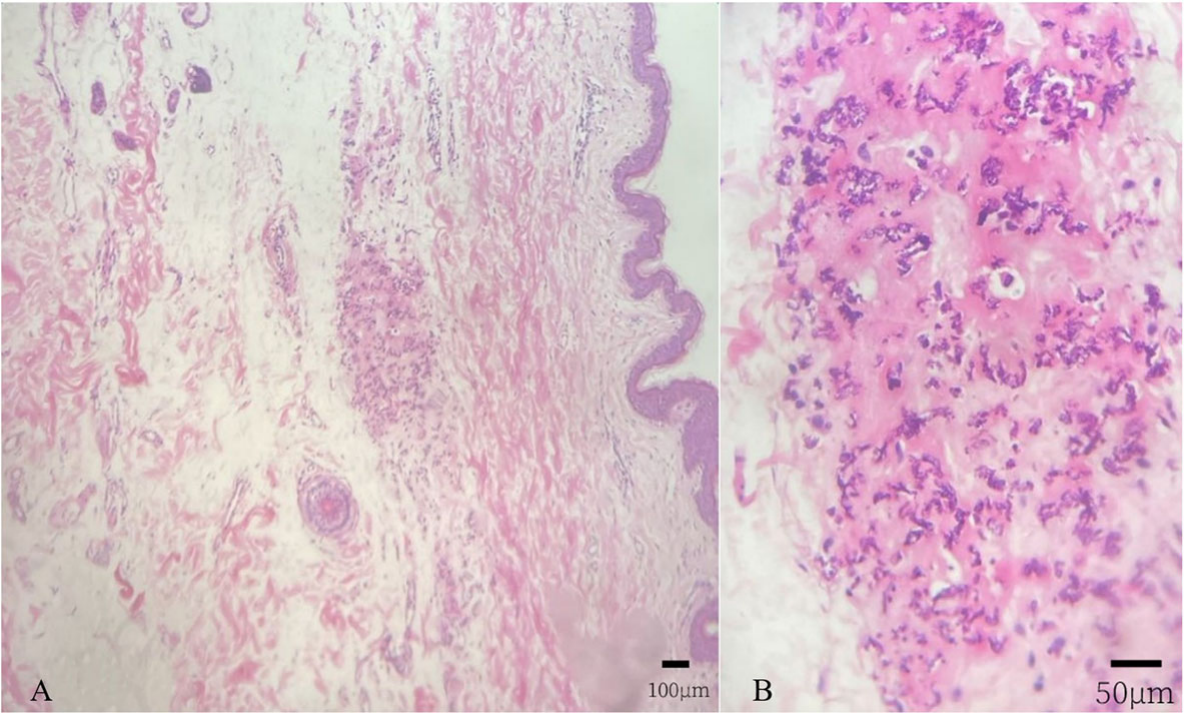
Neck skin tissue paraffin section. (Light microscope). (**A**) H&E staining× 10: The epidermis layer is normal; some blue stained structures are clustered in the dermis layer (Scale bar: 100 μm); (**B**) H&E staining× 40: The blue, irregular stained structures demonstrate calcinosis of the elastic fiber, the red stained areas are collagen fibers (Scale bar: 50 μm)

Primary diagnosis: (1) Secondary choroidal neovascularization (OU); (2) Pseudoxanthoma elasticum; (3) High myopia (OU); (4) Hypertension; (5) History of cerebral infarction. Due to her symptoms and manifestations, along with the appearance of her neck skin, which resembled ‘chicken skin’ (Fig. 4), we speculated that she should be further examined at the Department of Dermatology by tissue paraffin section and molecular pathology analyses (Fig. 5), and the diagnosis of PXE was then confirmed.

**Fig. 6:**
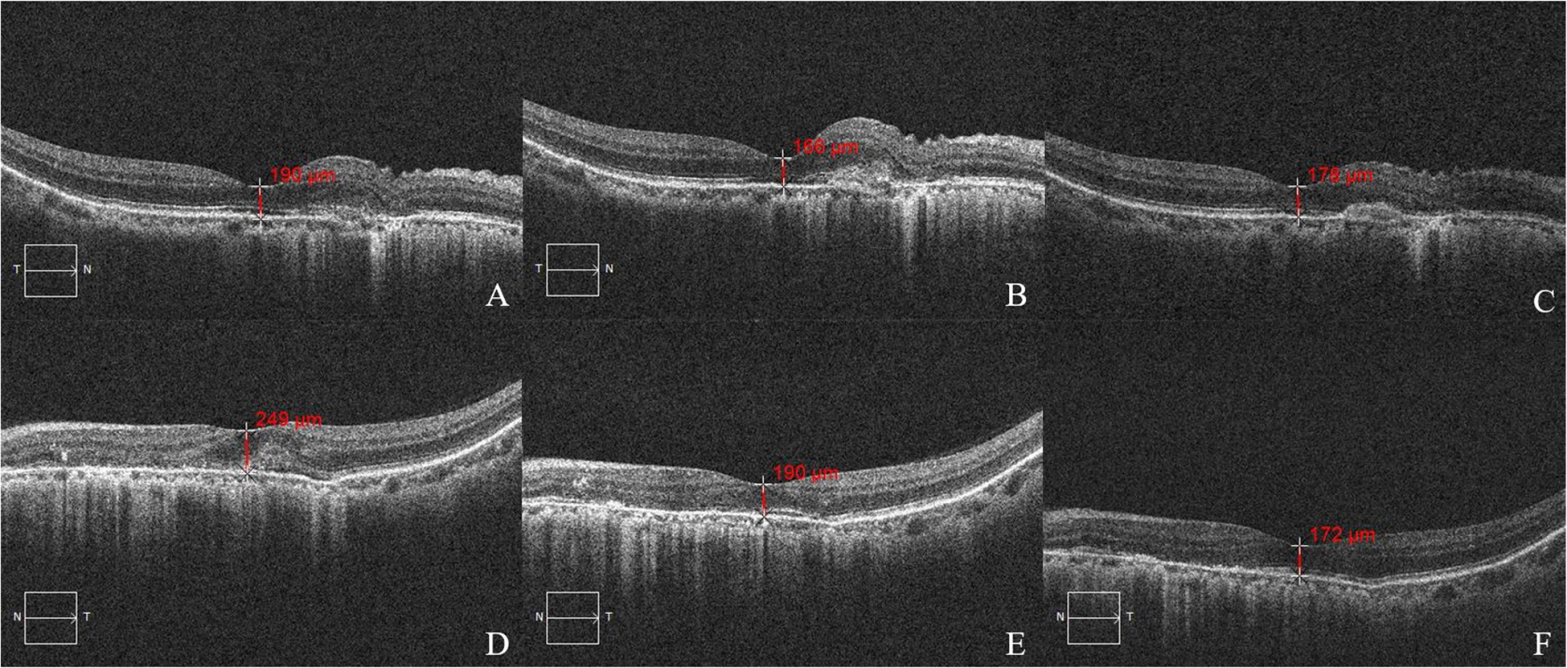
Dynamic changes in OCT of both eyes. (**A**)–(**C**) right eye, (**D**)–(**F**) left eye. (**A**), (**D**): 2020-9-14, (**B**), (**E**): 2020-10-7, (**C**), (**F**): 2020-11-23. (**A**)–(**D**): A hyperreflective lesion with fuzzy borders with an ‘absent’ of photoreceptor inner/outer segment junction was detected. (**E**)–(**F**): The hyperreflective lesion area was gradually shrinking. (**D**)–(**F**): Thinning of the central macular retinal thickness the left eye can be seen

To control the development of CNV, after excluding surgical contraindications, her treatment proceeded with the first administration of an intravitreal injection of Conbercept [3, 7, 14] (0.05 mL, 0.05 mL = 0.5 mg) (10 mg/ml, 0.2 ml, Chengdu Kanghong Biotechnologies Co., Ltd.; Chengdu, Sichuan, China) in the left eye on September 17, 2020. On postoperative day 1, the UCDVA was 0.04 and BCVA was 0.5^+ 2^ (− 7.00DS/− 0.50 DC × 110°), IOP = 18 mmHg. Twenty days after the intravitreal injection of left eye, the UCDVA was 0.01 and BCVA was 0.6^− 1^ (− 8.00DS/− 0.50 DC × 10°), IOP = 18 mmHg, OCT showed: CNV lesion area was gradually shrinking (Fig. 6). On October 22, 2020, a second intravitreal injection of Conbercept (0.05 mL, 0.05 mL = 0.5 mg) of the left eye was applied. On postoperative day 1, the UCDVA was 0.01 and BCVA was 0.6 (− 8.00DS), IOP = 17 mmHg. Then, on postoperative day 4, the UCDVA of 0.02 was achieved, and BCVA was 1.0 (− 7.00DS/− 0.75 DC × 90°), IOP = 17 mmHg. Meanwhile, on October 26, 2020, she received an intravitreal injection in the right eye for the first time. We established that on postoperative day 1, the UCDVA was 0.05 and BCVA was 0.6 (− 8.00DS/− 0.50 DC × 80°), IOP = 18 mmHg. On postoperative day 28, the UCDVA was 0.02 and BCVA was 0.8 (− 8.00DS), IOP = 17 mmHg. OCT: slight amelioration of CNV (Fig. 6). Levofloxacin eye drops (5 ml:24.4 mg, Santen Pharmaceutical Co., Ltd., Osaka, Japan) was used four times daily for one week after each surgery to prevent infection. The condition of both eyes was stable, with no significant change established at postoperative examination of OCT and she was satisfied with this outcome.

## Discussion and conclusions

Pseudoxanthoma elasticum (PXE) is an autosomal recessive disease, was first described in 1881, whose characteristic is the gradual calcification and degradation of elastic fibers, but it has been rarely reported as an autosomal dominant disease [1, 2]. PXE is characterized by late onset, slow progression, and considerable inter- and intra-family heterogeneity [15].

PXE is manifested by skin, cardiovascular, and oracular symptoms [2]. Skin symptoms are usually associated with the appearance of yellowish papules around the neck, the armpits, on the inner side of the elbow, the groins, and on the back of the knees. With these papules, the skin of the patient seems similar to chicken skin [16]. The most severe ocular manifestation, however, is observed at the late stages of the disease, which could lead to blindness [17]. AS are representative accompaniment of PXE, with an approximate incidence rate ranging from 1/100,000 to 1/20,000; the critical age range with the highest frequency of the condition is between 20 and 30 years [18]. AS represent radial streaks with a red, brown, or grey color, emitted from the optic disc to the equatorial boundary of both eyes [18, 19]; they have similar lengths in both eyes and elongate progressively with age [20]. The main pathology of AS is the calcification and fracture of Bruch’s membrane (BM). The elastin layer (EL) is a multi-layered lattice-like fibrous structure, which is the backbone providing the stability of BM. The calcification and degradation of EL is the internal factor which leads to the development of AS [21]. However, in this case, the patient had retinal and choroidal atrophy, with BM thinning and degradation which had progressively weakened its capacity for resistance to the action of external forces. We identified this influence as the external factor for the development of AS. In addition, our patient had high myopia, which might have been related to the formation of AS. The BM fracture disrupts the blood-retinal barrier, making it difficult for the vascular endothelial growth factor (VEGF) released by the retinal pigment epithelium (RPE) to reach the choroid. In turn, this affects the choroidal blood circulation, reducing the intrinsic choroidal capillary blood flow, which results in the lack of nutrients, accumulation of toxic substances, and activation of the inflammatory response. This outcome leads to the formation of CNV, which can grow between the RPE and photoreceptor layers through the BM fissure. When the lesion extends to the macula area, the occurring hemorrhage and exudation cause a dramatic loss of visual acuity [19, 20, 22]. We effectively ablated CNV, which reduced bleeding and exudation, and controlled the disease progression with anti-VEGF drugs. AS formation occurs not only in PXE, but also in other diseases, such as Marfan syndrome, β-thalassemia, and acromegaly [11, 23].

The fundus changes of PXE exerted a significant impact on the visual function and facilitated the diagnosis of this case. The skin lesions progress slowly and have minimal impact on daily life and can thus be easily overlooked. However, superficial skin lesions are conducive to a pathological examination for achieving a clear diagnosis. Moreover, the favorable combination of these two manifestations can considerably improve the diagnosis rate of PXE. We report a case of a patient with markedly improved BCVA after treatment, whose OCT examination showed regression of CNV. Such typical PXE with ocular manifestations and cutaneous manifestations is rare [19, 24]. However, unfortunately, our patient was not subjected to a genetic test. The incidence of PXE is extremely low, with no effective cure, targeted symptomatic treatment can effectively retard the disease progression and improve visual function [8, 11, 17]. The aim of this paper was to deepen the existing knowledge and understanding of PXE, so as to increase the accuracy of its diagnosis, prevent missed diagnosis, and provide more effective treatment for this disease.

Here, we made a clear diagnosis based on the observed fundus changes and the dermatopathological examination results. Furthermore, in the therapy of this case, anti-VEGF drugs effectively treated secondary CNV caused by AS of PXE. To the best of our knowledge, no publications are available on cases in which a vision loss and the normal visual function can be reverted by intravitreal injection with Conbercept [8–13]. This article and the effective treatment applied in this case may be a starting point of an effective therapeutic method of PXE with ocular manifestations and visual function impact.

## Data Availability

All data generated during this study are included in this published article.

## Abbreviations

AS: angioid streaks
BCVA: best corrected visual acuity
BM: Bruch’s membrane
CF: counting fingers
CNV: choroidal neovascularization
EL: elastin layer
FFA + ICGA: fluorescein angiography combined with indocyanine green angiography
Ig: immunoglobulin
IOP: intraocular pressure
OCT: optical coherence tomography
PlGF: placental growth factors
PXE: pseudoxanthoma elasticum
RPE: retinal pigment epithelium
UCDVA: uncorrected distance visual acuity
VEGF: vascular endothelial growth factor
VEGFR-1: growth factor receptor-1
VEGFR-2: vascular endothelial growth factor receptor-2
